# Oxytocin in Uniject Disposable Auto-Disable Injection System versus Standard Use for the Prevention of Postpartum Hemorrhage in Latin America and the Caribbean: A Cost-Effectiveness Analysis

**DOI:** 10.1371/journal.pone.0129044

**Published:** 2015-06-09

**Authors:** Andrés Pichon-Riviere, Demián Glujovsky, Osvaldo Ulises Garay, Federico Augustovski, Agustin Ciapponi, Magdalena Serpa, Fernando Althabe

**Affiliations:** 1 Department of Health Technology Assessment and Economic Evaluation, Institute for Clinical Effectiveness and Health Policy (IECS). Buenos Aires, Argentina; 2 Maternal and Child Health Integrated—Program (MCHIP)—PATH, Washington, D. C., United States of America; 3 Mother and Child Health Research Department, Institute for Clinical Effectiveness and Health Policy (IECS). Buenos Aires, Argentina; Kingston University London, UNITED KINGDOM

## Abstract

Postpartum hemorrhage (PPH) is a leading cause of maternal death. Despite strong evidence showing the efficacy of routine oxytocin in preventing PPH, the proportion of women receiving it after delivery is still below 100%. The Uniject injection system prefilled with oxytocin (Uniject) has the potential advantage, due to its ease of use, to increase oxytocin utilization rates. We aimed to assess its cost-effectiveness in Latin America and the Caribbean (LAC). We used an epidemiological model to estimate: a) the impact of replacing oxytocin in ampoules with Uniject on the incidence of PPH, quality-adjusted life years (QALYs) and costs from a health care system perspective, and b) the minimum increment in oxytocin utilization rates required to make Uniject a cost-effective strategy. A consensus panel of LAC experts was convened to quantify the expected increase in oxytocin rates as a consequence of making Uniject available. Deterministic and probabilistic sensitivity analyses were performed. In the base case, the incremental cost of Uniject with respect to oxytocin in ampoules was estimated to be USD 1.00 (2013 US dollars). In the cost-effectiveness analysis, Uniject ranged from being cost-saving (in 8 out of 30 countries) to having an incremental cost-effectiveness ratio (ICER) of USD 8,990 per QALY gained. In most countries these ICERs were below one GDP per capita. The minimum required increment in oxytocin rates to make Uniject a cost-effective strategy ranged from 1.3% in Suriname to 16.2% in Haiti. Switching to Uniject could prevent more than 40,000 PPH events annually in LAC. Uniject was cost-saving or very cost-effective in almost all countries. Even if countries can achieve only small increases in oxytocin rates by incorporating Uniject, this strategy could be considered a highly efficient use of resources. These results were robust in the sensitivity analysis under a wide range of assumptions.

## Introduction

Postpartum hemorrhage (PPH) is the leading cause of maternal death worldwide, accounting for approximately 127,000 deaths per year.[1–5] Besides its death toll, severe PPH is associated with prolonged hospital stay, admission to intensive care unit and higher health care cost.

The prophylactic administration of oxytocin, either alone or as part of active management of the third stage of labor (AMTSL), has been proven effective to prevent PPH and is strongly recommended as standard care in all deliveries.[6–8] Although there is strong evidence showing the efficacy of oxytocin/AMTSL in preventing up to 60% of PPH cases, its use in real-life settings and thus its effectiveness is suboptimal and heterogeneous. The proportion of women who receive oxytocin for prevention of PPH after delivery is still below 100%.[9–14]

BD Uniject SCF is a single-dose, disposable auto-disable pre-filled injection device that simplifies parenteral delivery of drugs, including oxytocin,[15] which makes it suitable for use by unskilled individuals or in settings with scarce personnel. As the efficacy of oxytocin administered through using ampoules and syringes (referred to as “standard oxytocin”) or through Uniject (referred to as “Uniject”) is similar, the main advantage of Uniject would be its potential, due to its ease of use,[16–18] to increase the proportion of women who receive oxytocin for prevention of PPH after delivery.

As economic considerations to prioritize resource allocation decisions are being increasingly accepted in Latin America, cost-effectiveness information could be a valuable tool to decide on the incorporation of technologies to health benefit packages or to inform price negotiations. [19,20]

Our objective was to perform a full economic evaluation that compared the cost-effectiveness of the standard practice (oxytocin in ampoules administered by health care providers in “syringe + needles”) vs. switching to Uniject to administer oxytocin in Latin America and the Caribbean (LAC).

## Material and Methods

As a general guidance to inform reporting, we used the Consolidated Health Economic Evaluation Reporting Standards (CHEERS) statement.[21]

Our target population were all women giving birth in a health facility a calendar year in each of the LAC countries. As in LAC countries home births represent less than 9%,[22] and usually these deliveries are not under the health care system control and influence, we focused our analysis only on the potential impact of introducing Uniject for deliveries in health facilities in LAC countries. Our study adopted a healthcare-sector perspective.

We compared two strategies: a) standard use of prophylactic oxytocin in ampoules administered in “syringe + needles” for prevention of PPH (10 IU IM or 5 IU IV); and b) Uniject as the injection system to administer oxytocin (10 IU per dose, intramuscular). We chose current practice as the comparator, as recommended by most economic evaluation guidelines,[23,24] since we considered it a more appropriate approach than the option of the “null” comparator used by other initiatives.[25]

We compared the progress of a hypothetical cohort of women giving birth in each LAC country using a lifetime horizon. Study outcomes for each strategy included PPH events, post-delivery hysterectomies, maternal deaths, LYs and QALYs. Two groups of parameters were included: global parameters, assumed not to vary by country (Table 1), and country-specific parameters (Table 2).

**Table 1:** Global parameters: Base case values, ranges used in the sensitivity analysis and data sources.

Parameters	Value & range	Source
Probability of PPH without oxytocin	0.12 (0.10–0.14)	[5,9,26]
Conditional probability of severe PPH (given PPH)	0.18 (0.15–0.21)	[1,9,26]
Risk of hysterectomy due to severe PPH	0.03 (0.02–0.05)	[14,26]
Case fatality ratio for home births (vs CEmONC facilities)	2.26 (1.66–7.19)	[27,28]
Case fatality ratio for BEMONC facilities (vs CEmONC)	1.88 (1.25–2.28)	[27]
Relative risk of PPH when receiving oxytocin	0.50 (0.43–0.57)	[29]
Discount rate	5% (0%- 10%)	[23]
QALY hysterectomy	0.99 (0.95–1.00)	[30]
Incremental cost of Uniject^†^ (US dollars of 2013)	$1.00 ($0.50–$1.50)	[31–33]
Oxytocin effectiveness^Ω^ (% gap reduction)	30.23% (12.03%-53.75%)	DELPHI Panel

Notes: PPH: postpartum hemorrhage; CEmONC facilities: facilities with comprehensive emergency obstetric and newborn care; BEmONC facilities: facilities with basic emergency obstetric and newborn care; QALY: quality-adjusted life years

^†^Incremental cost of Uniject relative to the traditional use of oxytocin in ampules

^Ω^Expressed as the proportion of the gap (between the ideal of 100% of women receiving oxytocin and the proportion currently receiving it) which could potentially be reduced by using Uniject instead of oxytocin in ampules.

For the probabilistic sensitivity analysis (PSA) the “probability of PPH without oxytocin”, the “conditional probability of severe PPH (given PPH)”, the “Risk of hysterectomy due to severe PPH”, the “Relative risk of PPH when receiving oxytocin” and the “Oxytocin effectiveness (% gap reduction)” were assumed to follow a Beta distribution. Alpha and beta parameters were approximated considering the mean the main estimate and as standard deviation the 10% of the mean value. The “incremental cost of Uniject” was assumed to follow a Gamma distribution. Alpha and beta parameters were approximated in these cases considering the mean as the main estimate and as standard deviation the 25% of the mean value.

**Table 2:** Country-specific parameters: Base case values, ranges used in sensitivity analysis and source of data.

Country	Annual deliveries [34]	Age at delivery (years)	Skilled birth attendance (%) [35]	Proportion CEmONC (%) [35,36]	Oxytocin use (%) [14,37,38]	Maternal mortality rate (p/100,000) [39]	Proportion of deaths due to PPH (%)	Life expectancy at age of delivery (years) [40]	PPH episode cost (non-severe) US$ 2013	PPH episode cost (severe) US$ 2013	Exch. Rate 2013 (US$ 1) [41]	GDPpp (thousands US$ 2013) [41]
Argentina	694	25.5 (23.0–28.1) [42]	99	80 ^B^	71.1 (57.7–88.4)	77.0 (67.0–87.)	10.00 (5.6–14.9) [43–45]	81.0 (77.0–85.0)	$76.8 (57.6–96.0) ^C^	$978.6 (733.9–1,223.2) ^C^	$ 5.2	$ 12.0
Bahamas	1,5	21.6 (19.5–23.8) ^A^	99	80 ^B^	71.6 (57.6–89.1)	47.0 (28.0–75.)	16.05 (12.0–20.1) ^A^	79.0 (75.0–83.0)	$151.6 (113.7–189.5) ^C^	$2,103.7 (1,577.8–2,629.6) ^C^	$ 1.0	$ 23.5
Barbados	3	21.6 (19.5–23.8) ^A^	99	80 ^B^	71.6 (57.6–89.1)	51.0 (19.0–140.)	9.70 (7.3–12.1) [1]	82.0 (78.0–86.0)	$108.7 (81.5–135.8) ^C^	$1,450.5 (1,087.9–1,813.1) ^C^	$ 2.0	$ 16.8
Belize	8	21.6 (19.5–23.8) ^A^	95	80 ^B^	68.7 (55.3–85.5)	53.0 (33.0–88.)	16.05 (12.0–20.1) ^A^	80.0 (76.0–84.0)	$36.1 (27.1–45.1) ^C^	$357.9 (268.4–447.4) ^C^	$ 2.0	$ 4.6
Bolivia	263	21.1 (19.0–23.2) [42]	71	30 ^B^	51.4 (41.3–63.9)	190.0 (130.0–290.)	15.40 (11.6–19.3) D	74.0 (70.0–78.0)	$26.4 (19.8–33.0) ^C^	$211.3 (158.4–264.1) ^C^	$ 6.7	$ 2.7
Brazil	3,023,000	21.6 (19.5–23.8) [42]	97	80 ^B^	74.2 (59.6–92.2)	56.0 (36.0–85.)	10.90 (8.2–13.6) [43]	79.0 (75.0–83.0)	$41.8 (31.4–52.3) ^C^	$459.3 (344.5–574.1) ^C^	$ 1.7	$ 12.3
Chile	241,5	21.6 (19.5–23.8) [42]	99	80 ^B^	71.6 (57.6–89.1)	25.0 (21.0–29.)	6.53 (4.9–8.2) [2]	84.0 (80.0–88.0)	$96.6 (72.5–120.8) ^C^	$1,273.6 (955.2–1,591.9) ^C^	$ 516.0	$ 16.3
Colombia	914	21.6 (19.4–23.8) [42]	96	80 ^B^	69.5 (55.9–86.4)	92.0 (80.0–100.)	17.70 (13.3–22.1) ^D^	84.0 (80.0–88.0)	$51.2 (38.4–64.0) ^C^	$588.7 (441.5–735.9) ^C^	$ 1,954.0	$ 8.2
Costa Rica	73	21.6 (19.5–23.8) ^A^	99	80 ^B^	71.6 (57.6–89.1)	40.0 (15.0–31.)	15.70 (11.8–19.6) [43]	82.0 (78.0–86.0)	$63.6 (47.7–79.4) ^C^	$776.7 (582.5–970.8) ^C^	$ 532.3	$ 10.4
Cuba	112	21.6 (19.5–23.8) ^A^	99	80 ^B^	71.6 (57.6–89.1)	73.0 (60.0–87.)	4.40 (3.3–5.5) [43]	81.0 (77.0–85.0)	$40.9 (30.7–51.2) ^C^	$427.0 (320.3–533.8) ^C^	$ 1.0	$ 5.4
Dominican Rep.	216	21.6 (19.5–23.8) ^A^	98	80 ^B^	70.9 (57.0–88.2)	115.0 (100.0–210.)	12.65 (9.5–15.8) [43]	80.0 (76.0–84.0)	$41.4 (31.0–51.7) ^C^	$438.6 (328.9–548.2) ^C^	$ 41.8	$ 5.8
Ecuador	299	21.6 (19.5–23.8) ^A^	99	80 ^B^	66.6 (53.6–82.9)	110.0 (62.0–180.)	29.40 (22.1–36.8) [43]	80.0 (76.0–84.0)	$41.5 (31.1–51.9) ^C^	$439.1 (329.3–548.8) ^C^	$ 1.0	$ 5.6
El Salvador	126	21.6 (19.5–23.8) ^A^	84	50 ^B^	60.8 (48.9–75.6)	81.0 (55.0–120.)	16.05 (12.0–20.1) [10]	78.0 (74.0–82.0)	$32.5 (24.4–40.6) ^C^	$303.0 (227.3–378.8) ^C^	$ 1.0	$ 3.9
Grenada	2	21.6 (19.5–23.8) ^A^	99	80 ^B^	71.6 (57.6–89.1)	24.0 (15.0–38.)	16.05 (12.0–20.1) ^A^	79.0 (75.0–83.0)	$53.6 (40.2–67.1) ^C^	$623.0 (467.3–778.8) ^C^	$ 2.7	$ 7.8
Guatemala	467	19.9 (17.9–21.9) [46]	51	30 ^B^	36.9 (29.7–45.9)	120.0 (110.0–140.)	58.10 (43.6–72.6) [11]	78.0 (74.0–82.0)	$28.4 (21.3–35.6) ^C^	$246.3 (184.7–307.9) ^C^	$ 8.4	$ 3.4
Guyana	14	20.7 (18.6–22.8) ^A^	83	50 ^B^	60.1 (48.3–74.7)	280.0 (180.0–430.)	16.05 (12.0–20.1) ^A^	70.0 (66.0–74.0)	$32.5 (24.4–40.6) ^C^	$300.9 (225.7–376.2) ^C^	$ 209.6	$ 3.9
Haiti	266	20.1 (18.1–22.1) ^A^	26	30 ^B^	18.8 (15.1–23.4)	315.0 (210.0–610.)	16.05 (12.0–20.1) ^A^	70.0 (66.0–74.0)	$17.9 (13.4–22.4) ^C^	$87.4 (65.6–109.3) ^C^	$ 40.8	$ 0.8
Honduras	203	22.2 (20.0–24.4) [42]	67	30 ^B^	48.5 (39.0–60.3)	100.0 (64.0–160.)	47.06 (35.3–58.8) [12]	79.0 (75.0–83.0)	$24.5 (18.4–30.6) ^C^	$182.6 (136.9–228.2) ^C^	$ 20.1	$ 2.3
Jamaica	51	21.6 (19.5–23.8) ^A^	96	80 ^B^	69.5 (55.9–86.4)	110.0 (77.0–170.)	16.05 (12.0–20.1) ^A^	79.0 (75.0–83.0)	$41.3 (31.0–51.6) ^C^	$437.7 (328.3–547.1) ^C^	$ 91.9	$ 5.6
Mexico	2,217,000	21.6 (19.5–23.8) [42]	94	50 ^B^	71.6 (57.6–89.1)	50.0 (44.0–56.)	24.30 (18.2–30.4) [43]	80.0 (76.0–84.0)	$74.7 (56.0–93.3) ^C^	$941.3 (705.9–1,176.6) ^C^	$ 12.3	$ 11.0
Nicaragua	138	21.6 (19.5–23.8) [46]	74	30 ^B^	54.1 (43.5–67.3)	95.0 (54.0–170.)	16.05 (12.0–20.1) [13]	78.0 (74.0–82.0)	$22.5 (16.9–28.1) ^C^	$148.7 (111.6–185.9) ^C^	$ 24.7	$ 1.8
Panama	70	21.6 (19.5–23.8) [42]	91	50 ^B^	65.9 (52.9–81.9)	92.0 (75.0–110.)	16.40 (12.3–20.5) [43]	82.0 (78.0–86.0)	$72.4 (54.3–90.5) ^C^	$904.7 (678.6–1,130.9) ^C^	$ 1.0	$ 11.1
Paraguay	156	21.6 (19.5–23.8) [42]	97	80 ^B^	71.1 (57.2–88.5)	99.0 (60.0–160.)	25.40 (19.1–31.8) [43]	80.0 (76.0–84.0)	$35.6 (26.7–44.5) ^C^	$355.7 (266.8–444.6) ^C^	$ 4,043.7	$ 4.5
Peru	154	21.9 (20.0–22.9) [42]	83	50 ^B^	65.6 (52.7–81.6)	67.0 (42.0–110.)	50.00 (37.5–62.5) [43]	80.0 (76.0–84.0)	$45.7 (34.3–57.2) ^C^	$502.8 (377.1–628.5) ^C^	$ 2.9	$ 7.1
Saint Lucia	3	21.6 (19.5–23.8) ^A^	99	80 ^B^	71.6 (57.6–89.1)	35.0 (22.0–54.)	16.05 (12.0–20.1) ^A^	80.0 (76.0–84.0)	$52.5 (39.4–65.6) ^C^	$616.9 (462.7–771.2) ^C^	$ 2.7	$ 7.6
St. Vincent & Grenadines	2	21.6 (19.5–23.8) ^A^	99	80 ^B^	71.6 (57.6–89.1)	48.0 (30.0–78.)	16.05 (12.0–20.1) ^A^	78.0 (74.0–82.0)	$47.2 (35.4–59.0) ^C^	$537.9 (403.4–672.4) ^C^	$ 2.7	$ 6.7
Suriname	10	21.6 (19.5–23.8) [42]	90	50 ^B^	65.1 (52.4–81.0)	130.0 (89.0–190.)	36.00 (27.0–45.0) [43]	81.0 (77.0–85.0)	$61.5 (46.1–76.9) ^C^	$749.1 (561.8–936.3) ^C^	$ 3.3	$ 9.5
Trinidad & Tobago	20	21.6 (19.5–23.8) [42]	98	80 ^B^	70.9 (57.0–88.2)	46.0 (26.0–84.)	2.80 (2.1–3.5) [43]	76.0 (72.0–80.0)	$122.3 (91.7–152.8) ^C^	$1,660.7 (1,245.5–2,075.8) ^C^	$ 6.4	$ 20.1
Uruguay	15	21.6 (19.5–23.8) [42]	99	80 ^B^	71.6 (57.6–89.1)	29.0 (21.0–39.)	4.40 (3.3–5.5) [43]	82.0 (78.0–86.0)	$99.1 (74.3–123.9) ^C^	$1,315.2 (986.4–1,643.9) ^C^	$ 20.0	$ 15.3
Venezuela	158	21.6 (19.5–23.8) [42]	95	80 ^B^	68.7 (55.3–85.5)	92.0 (78.0–110.)	17.01 (12.8–21.3) [2,43] ^D^	81.0 (77.0–85.0)	$69.0 (51.8–86.3) ^C^	$858.9 (644.2–1,073.7) ^C^	$ 6.5	$ 11.5

Notes:

Abbreviations: Proportion CEmONC: proportion of births at facilities with comprehensive emergency obstetric and newborn care; PPH: postpartum hemorrhage; Exch. Rate: Exchange rate; GDPpp: gross domestic product per capita;

^A^: average from other countries (see text);

^B^: estimation based on data from Argentina, Bolivia, El Salvador, and Honduras and references 4 and 5 (see text);

^C^: see methods in manuscript (cost section);

**^D^:** Data from databases of Ministries of Health (personal communication).

Probability distributions: for the probabilistic sensitivity analysis (PSA) we modeled the variability of Proportion CEmONC (%), Oxytocin use (%) and Proportion of deaths due to PPH (%) using a Beta distribution. Alpha and beta parameters were approximated considering the mean as main estimate and as standard deviation the 10% of the mean value. The costs of severe and non-severe PPH were assumed to follow a Gamma distribution. Alpha and beta parameters were approximated in these cases considering the mean as the main estimate and as standard deviation the 25% of the mean value.

Future health outcomes were discounted at an annual rate of 5% following the recommendation of most LAC guidelines.[23] It was assumed that a PPH episode would have no implications on long-term health care costs.

### Measures of effectiveness and analytical methods

One of the main limitations in accurately estimating the cost-effectiveness of Uniject versus standard oxytocin is that there is no high-quality evidence about the relative effectiveness of Uniject (i.e., rate of change in the use of oxytocin when Uniject is made available). Thus, two different approaches were followed to assess the cost-effectiveness of the Uniject device:

1) Standard cost-Effectiveness analysis: a standard cost-effectiveness analysis was performed using an estimate of the relative effectiveness of Uniject obtained from a consensus panel of recognized experts in Latin-American maternal health convened through a modified Delphi panel methodology as part of this study.[47]

The expert panel estimated the expected increase in oxytocin coverage rates if standard oxytocin were replaced with Uniject without any other intervention or educational measure tending to increase the use of oxytocin. After two rounds in which they received feedback on their responses, the experts estimated a mean expected effect and a minimum and maximum effect to be explored in the sensitivity analysis.

Cost-effectiveness was evaluated using the incremental cost-effectiveness ratio (ICER). ICERs are calculated as the ratio the difference in costs (Δ costs) and the difference in benefits (Δ consequences) of two alternatives. Costs included the costs of implementing the strategy (i.e., differential cost of the Uniject device) minus any medical costs averted (i.e., events of PPH). The change in consequences was the difference in health outcomes between the Uniject and the ampoule arm (expressed as life years [LYs] or QALYs gained). Thus, the ICER reflected the additional cost for each additional unit of outcome obtained as a result of using Uniject.

2) Threshold analysis: in order to avoid relying solely on the estimation of effectiveness provided by the expert panel, we estimated the minimum change in the rate of oxytocin use that would be necessary for Uniject to be cost-effective, as judged by the widely used World Health Organization (WHO) decision rule: if an intervention incremental cost per healthy year gained is equal or below one gross domestic product (GDP) per capita, it is cost-effective.[48,49] The objective of this analysis was to answer the following question: “How much should Uniject increase the rate of prophylactic oxytocin use to be considered a cost-effective intervention?” With this information, a decision-maker can judge whether this required increase in the use of oxytocin is realistic and can be obtained in his/her context by switching to Uniject.

### Selection of information sources and parameter incorporation

A literature review limited to the last 15 years was conducted in general and specialized databases: MEDLINE, CENTRAL, Cochrane Pregnancy and Childbirth Group’s Trials Register, Latin American and Caribbean Health Science Information, WHO and Pan American Health Organization (PAHO) databases, nongovernmental organizations (NGOs) and other organizations known to be active in the maternal health field to find datasets not captured by bibliographic searches (such as United Nations, Department of Economic and Social Affairs; Global Health Observatory Data Repository of the World Health Organization; World Bank Life expectancy tables; National health network hospitals in El Salvador, Guatemala, Honduras, and Nicaragua). In addition, ministerial databases of LAC countries were reviewed. The search strategy included the following MeSH and free-text terms for PPH and its causes: ‘postpartum hemorrhage’, ‘hemorrhage/and (pregnant* or postpartum* or postpartum or post partal)’, ‘epidemiological data’, ‘Maternal Health Services’, ‘hospital information system’, ‘medical information system’, ‘facility’, ‘maternal mortality’, ‘maternal death’, ‘oxytocin’, ‘third stage of labor’.

An evidence hierarchy was used in order to select the most appropriate sources to populate the model (i.e., population-based observational studies for epidemiological or resource use data, experimental/RCT evidence for comparative effects). Prospective designs were prioritized.

### Resource use and Costs

The incremental cost of Uniject was estimated as the difference in cost between Uniject and 10 IU of oxytocin in ampoules plus the costs of disposable syringes and needles.

Transportation cost and personnel salaries were not included as these costs did not vary significantly between Uniject and standard oxytocin in other studies [32,33]. We included an adjustment for the wastage rate of ampoules and the costs due to the increase in space required for storage of Uniject. Intervention start-up costs were not included so that all interventions were evaluated and compared as if operating under steady-state conditions.

All costs were expressed in 2013 US dollars (US$) according to the current exchange rates published by the International Monetary Fund (IMF).[41]

#### Cost of events

Two clinical events were included in the model: non-severe PPH (blood loss of 500–1000 mL) and severe PPH (blood loss of >1000 mL). Both were estimated following a micro-costing approach, using a list of resources and utilization rates identified by experts in the field. Specifically for hospital-stay costs, the main driver of the total costs, we used the methodology proposed by WHO-CHOICE[50] with values updated to 2013, except for Brazil where inpatient costs were obtained from Longo et al and updated to 2013 using the local consumer price index.[41,51] The WHO CHOICE methodology consists in predicting hospital-stay costs for each country using a logarithmic regression model composed of macro level variables such as the GDP per capita, Purchase Power Parities (PPP) and occupancy rates. Unit costs missing in specific countries were estimated following an approach similar to Johns [52] and Goldhaber-Fiebert [53] in which an average international scenario expressed in international dollars (I$) and based on the best available information from the remaining LAC countries is converted to US$ according to each country’s exchange rates. For non-tradable goods, we used Purchasing Power Parities (PPP) from World Bank and for tradable goods, we assumed an exchange rate of 1US$ = 1I$. When needed, unit costs were adjusted using Consumer Price Indexes (CPI) to reflect the base case year of costs. Data on prices were obtained from the World Outlook Database of IMF.[41]

### Model description

As the study addresses an acute and static health problem and intervention, an epidemiological model that could capture the most relevant events and costs of the two interventions was designed. The model estimated the impact of replacing standard oxytocin with Uniject on the incidence of PPH, on the associated health consequences of PPH events and on health care costs. Excel (Microsoft Professional Edition 2010) with Visual Basic Macros (Microsoft Visual Basic 7.0) was selected as the model platform. The International Society for Pharmacoeconomics and Outcomes Research criteria for model development and reporting were applied.[54]

**Fig 1:**
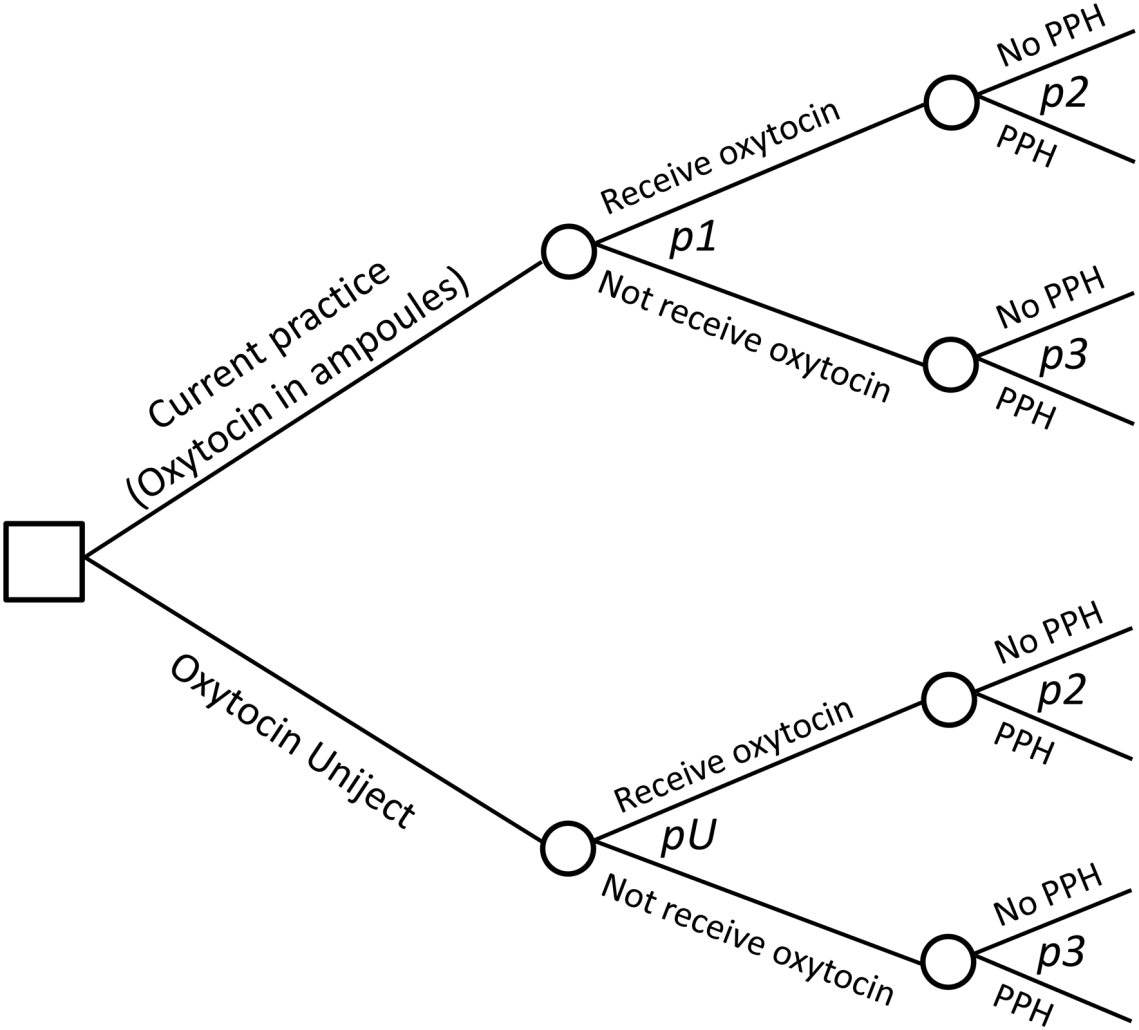
Schematic representation of model structure. Notes: Two strategies are compared: oxytocin in ampoules plus syringes and needles (Current practice branch) vs replacing current practice with Uniject (Oxytocin Uniject branch). The only differential effect of Uniject is modeled as a change (increase) in the probability of receiving oxytocin (pU in the Oxytocin Uniject branch). All other probabilities (having PPH in those women not receiving oxytocin—p2, and in those who do receive oxytocin-p3) are the same in both strategies.

The model is based on the assumption that the baseline risk of PPH (i.e., the woman’s risk of PPH when oxytocin is not administered and without mediating any additional preventive intervention) is similar for all deliveries in all countries.[55] This baseline risk is then modified by the current rates of oxytocin use in each country. The Uniject effect being modeled is its potential to increase the proportion of women receiving oxytocin. See Fig 1 for a schematic representation of the model structure.

To estimate the minimum increase in oxytocin use that would be needed for Uniject to become a cost-effective intervention, we initially estimated the number of PPH events needed to be prevented as:

PPH_avoid = Cost_intervention  (Cost_PPH + QALY_lost * CE_Thr) (1)

Where *PPH_avoid* is the number of PPH events that would be needed to be prevented; *Cost_intervention* is the total incremental cost of replacing standard oxytocin with Uniject in the places where the intervention is planned; *Cost_PPH* is the mean cost of each PPH episode; QALY_lost is the mean number of QALY lost for each PPH episode (these last two values were obtained from the calibrated model for each country); and *CE_Thr* is the cost-effectiveness threshold (assumed to be one GDP per capita per QALY in the base case).

Then, the new risk of PPH that would be necessary to achieve was estimated as:

R_PPH_target = R_PPH_country – (PPH_avoid/total_deliveries) (2)

Where *R_PPH_target* is the risk of PPH that would be necessary to achieve for Uniject to be cost-effective; *R_PPH_country* is the risk of PPH at the country level; *PPH_avoid* is the number of PPH events that would be needed to be prevented; and *total_deliveries* is the total number of deliveries.

And finally, the new oxytocin coverage rate at the country level that would be necessary to obtain was estimated as:

Target_Ox  =(R_PPH_target - R_PPH_basal) (R_PPH_basal*( RR_Ox_protec – 1))  (3)

Where *Target_Ox* is the new oxytocin coverage rate at the country level that would be necessary to achieve for Uniject to be cost-effective; *R_PPH_target* is the risk of PPH that would be necessary to achieve; *R_PPH_basal* is the baseline risk of PPH; and *RR_Ox_protec* is the relative risk of PPH when receiving oxytocin.

### Internal validation and calibration

Internal testing and debugging were performed to check that the mathematical calculations were accurate and consistent with the specifications of the model. Calibration was carried out to ensure that the model adequately reflected the current situation in each country. This was performed comparing the PPH death rates predicted by the model with local health statistics. The case fatality rates of PPH events were calibrated in each country in order to obtain PPH death rates consistent with local statistics.

### Sensitivity analysis

A deterministic sensitivity analysis (DSA) was performed to estimate the univariate impact of inputs on results. The main model parameters were included in a probabilistic sensitivity analysis (PSA) where all inputs were varied simultaneously across 1,000 Monte Carlo simulations. Confidence intervals (95% CI) were estimated as the range between observations at percentiles 2.5 and 97.5 from the Monte Carlo simulations results.

## Results

### Study Parameters

Global parameters and country specific parameters are summarized in Tables 1 and 2, including base-case values, ranges, distributions used for the sensitivity analysis and data sources. Maternal mortality ratios were retrieved from the WHO report 2010,[56] the proportion of deaths due to PPH was obtained from published literature,[10–14,44,57] and data about annual deliveries was obtained from UN databases 2010.[58] In those cases where country specific data were not available, we used the median value obtained from the countries for which information was available.

The baseline incidence of PPH (without oxytocin) and the proportion of PPH that are severe were estimated from published literature.[1,5,9,26] We assumed that AMTSL reduced the rate of PPH by 50% based on Begley et al[7] and that this effect was mainly attributable to the use of oxytocin, as has been shown in a trial comparing AMSTL vs oxytocin alone.[7,59,60]

Data of oxytocin coverage rates in each country was obtained from Souza et al and from several reports from POPPHI.[10–14] We judged that data on oxytocin use obtained from Souza et al could overestimate the actual coverage rates as they come from a biased sample of selected tertiary care facilities.[14] For this reason, data from Souza et al were used as the maximal value. In order to obtain the base case value, data from Karolinski et al were used to adjust country data, assuming that the relative difference (0.72) between Souza and Karolinski for the case of Argentina could be extrapolated to the other countries. For the minimum oxytocin use value estimation, we assumed that it was equal to the difference between the maximum and base-case value.

#### Life years (LYs) and quality adjusted life years (QALYs)

Women surviving birth were assumed to have the life expectancy for women in their respective countries, and those dying from PPH were assumed to die at the mean age of delivery in each country. In order to derive QALYs, we reviewed studies that reported quality of life after surviving a hysterectomy during a woman´s lifetime. We used a value of 0.985 in order to incorporate the long-term quality of life decrements due to hysterectomy.[30]

#### Measure of effectiveness and costs of Uniject

Eight recognized experts in maternal and child health with extensive experience in LAC participated in the Delphi panel and completed the two rounds of consultations (see acknowledgments). The expert panel estimated that in settings with a baseline use of prophylactic oxytocin of 50%, Uniject might increase its use up to 64.1%, which implies a 28.1% reduction in the existing gap and to 86.5% in those settings with a baseline use of 80%, which implies a gap reduction of 32.3%. In order to obtain a measure of effectiveness of Uniject that could be applicable to all countries and, based on these results, we estimated that replacing standard oxytocin with Uniject would save 30.2% of the current gap in the oxytocin coverage rate (average of the gap reductions forecasted by the experts in the scenarios with a baseline use of prophylactic oxytocin of 50% and 80%). Based on the uncertainty ranges estimated by the expert panel, the range to be used in the sensitivity analysis was defined to be between 12.0% and 53.8%.

When analyzing the cost of oxytocin in ampoules, syringes, needles and Uniject, we found great variability among countries and uncertainty on international prices. Therefore, based on data from other studies [31–33] we assumed a cost of USD 0.25 for one ampoule of 10 IU of oxytocin plus one disposable syringe and one needle and a cost of USD 1.25 for Uniject, all resulting in an incremental cost of USD 1.00 for using oxytocin in Uniject instead of in ampoules. We analyzed two additional scenarios where the incremental cost of Uniject was set at USD 0.50 and USD 1.50 in order to contemplate the range of most likely values found in the international literature.

### Incremental costs and outcomes

In the 30 countries analyzed, the Uniject strategy showed a reduction in PPH events and deaths and an increase in QALYS. Uniject could prevent more than 40,000 PPH events annually, accounting for more than 4,000 LYs saved in LAC countries. The incremental QALYs per 1,000 institutional deliveries ranged from 0.02 to 0.71 (Table 3). In 27% of the countries, Uniject was cost-saving. In the remaining 22 countries, Uniject was associated with a net cost increment that ranged from $ 0.005 to $0.85 per delivery.

**Table 3:** Base case results and 95% confidence intervals from probabilistic sensitivity analysis, 2013US dollars, 5% discount rate.

Country	Threshold Analysis	Cost-Effectiveness Analysis					
	Minimum absolute incremental increase in oxytocin use required to make Uniject cost-effective at a threshold of 1 GDPPC per QALY	Cost difference per 1,000 institutional deliveries	Incremental QALYs per 1,000 institutional deliveries	Incremental cost-effectiveness ratio ($ per QALY)	Prob. of being cost saving	Prob. of being cost-effective at a threshold of 1 GDPPC^a^	Prob. of being cost-effective at a threshold of 3 GDPPC^b^
Argentina	3.38%	$ -223.20	0.11	Cost Saving	62%	98%	100%
	(1.7% to 6.2%)	(-$ 1,329 to $ 723)	(0.05 to 0.18)				
Bahamas	1.68%	$ -1,521.80	0.1	Cost Saving	97%	100%	100%
	(0.9% to 3.1%)	(-$ 3,664 to $ 117)	(0.04 to 0.19)				
Barbados	2.81%	$ -755.70	0.07	Cost Saving	89%	99%	100%
	(1.3% to 5.8%)	(-$ 2,422 to $ 391)	(0.01 to 0.15)				
Belize	8.32%	$ 528.50	0.12	4,585	3%	47%	94%
	(4.4% to 15.4%)	(-$ 14 to $ 1,152)	(0.05 to 0.20)	(-107 to 19,165)			
Bolivia	7.11%	$ 700.70	0.33	2,141	0%	64%	99%
	(3.8% to 12.9%)	($ 165 to $ 916)	(0.10 to 0.43)	(517 to 6,916)			
Brazil	5.01%	$ 500.60	0.07	6,676	8%	73%	99%
	(2.6% to 9.1%)	(-$ 152 to $ 1,142)	(0.03 to 0.14)	(-1,307 to 31,343)			
Chile	4.12%	$ -546.50	0.03	Cost Saving	82%	94%	99%
	(2.0% to 7.7%)	(-$ 2,076 to $ 508)	(0.01 to 0.05)				
Colombia	3.31%	$ 258.30	0.22	1,192	23%	99%	100%
	(1.7% to 6.0%)	(-$ 511 to $ 1,004)	(0.11 to 0.36)	(-1,873 to 7,077)			
Costa Rica	4.42%	$ 37.80	0.09	422	44%	95%	100%
	(2.3% to 8.0%)	(-$ 904 to $ 785)	(0.04 to 0.15)	(-8,187 to 14,386)			
Cuba	10.18%	$ 446.40	0.05	8,99	8%	32%	79%
	(5.3% to 18.5%)	(-$ 163 to $ 1,078)	(0.02 to 0.08)	(-2,385 to 34,339)			
Dominican Rep.	4.94%	$ 434.00	0.19	2,233	9%	88%	100%
	(2.5% to 9.3%)	(-$ 212 to $ 1,037)	(0.08 to 0.36)	(-894 to 9,612)			
Ecuador	2.91%	$ 329.30	0.48	681	17%	100%	100%
	(1.4% to 5.9%)	(-$ 353 to $ 934)	(0.22 to 0.82)	(-614 to 3,224)			
El Salvador	8.02%	$ 593.10	0.16	3,673	1%	53%	97%
	(4.1% to 14.0%)	($ 56 to $ 967)	(0.06 to 0.25)	(336 to 12,088)			
Grenada	6.79%	$ 217.20	0.06	3,773	27%	67%	97%
	(3.4% to 11.8%)	(-$ 663 to $ 935)	(0.03 to 0.10)	(-8,811 to 24,760)			
Guatemala	3.01%	$ 660.80	0.71	925	1%	99%	100%
	(1.6% to 5.0%)	($ 63 to $ 614)	(0.16 to 0.61)	(140 to 2,855)			
Guyana	3.45%	$ 594.80	0.52	1,143	2%	97%	100%
	(1.7% to 6.6%)	($ 20 to $ 995)	(0.18 to 0.76)	(50 to 4,171)			
Haiti	15.81%	$ 847.50	0.45	1,864	0%	6%	65%
	(7.9% to 32.1%)	($ 105 to $ 373)	(0.04 to 0.23)	(676 to 6,625)			
Honduras	5.78%	$ 734.60	0.52	1,415	0%	77%	99%
	(2.8% to 11.1%)	($ 166 to $ 867)	(0.13 to 0.66)	(363 to 4,677)			
Jamaica	4.50%	$ 435.20	0.23	1,884	9%	92%	100%
	(2.2% to 8.2%)	(-$ 242 to $ 1,030)	(0.10 to 0.41)	(-758 to 7,026)			
Mexico	2.82%	$ 5.10	0.14	36	46%	97%	100%
	(1.5% to 4.9%)	(-$ 1,083 to $ 830)	(0.06 to 0.24)	(-5,489 to 11,657)			
Nicaragua	15.02%	$ 780.00	0.17	4,468	0%	6%	60%
	(7.7% to 28.4%)	($ 248 to $ 1,001)	(0.05 to 0.25)	(1,444 to 13,712)			
Panama	2.54%	$ -114.10	0.2	Cost Saving	56%	99%	100%
	(1.3% to 4.4%)	(-$ 1,074 to $ 715)	(0.08 to 0.30)				
Paraguay	4.24%	$ 548.90	0.32 (0.13 to 0.56)	1,72	3%	91%	100%
	(2.1% to 8.2%)	(-$ 43 to $ 1,154)		(-105 to 6,747)			
Peru	2.30%	$ 513.20	0.32	1,612	7%	95%	99%
	(1.1% to 4.7%)	(-$ 141 to $ 956)	(0.07 to 0.53)	(-418 to 9,194)			
Saint Lucia	6.03%	$ 227.40	0.08	2,825	26%	76%	99%
	(3.0% to 11.0%)	(-$ 622 to $ 900)	(0.03 to 0.14)	(-6,357 to 20,598)			
St. Vincent & Grenadines	6.00%	$ 320.40	0.11	3,003	18%	77%	99%
	(3.0% to 11.1%)	(-$ 480 to $ 1,079)	(0.04 to 0.19)	(-3,123 to 15,909)			
Suriname	1.29%	$ 71.20	0.58	122	40%	100%	100%
	(0.6% to 2.5%)	(-$ 836 to $ 789)	(0.24 to 0.98)	(-1,193 to 2,304)			
Trinidad & Tobago	3.36%	$ -1,001.20	0.02	Cost Saving	90%	96%	100%
	(1.7% to 6.0%)	(-$ 2,874 to $ 340)	(0.01 to 0.04)				
Uruguay	4.24%	$ -594.30	0.02	Cost Saving	82%	92%	98%
	(2.2% to 7.8%)	(-$ 2,102 to $ 506)	(0.01 to 0.04)				
Venezuela	2.43%	$ -58.90	0.21	Cost Saving	52%	100%	100%
	(1.2% to 4.2%)	(-$ 1,132 to $ 767)	(0.09 to 0.34)				

CE: cost-effective; GDPPC: gross domestic product per capita; QALY: quality-adjusted life years; Prob.: probability.

^a^Refers to a threshold of 1 GDPPC per QALY;

^b^Refers to a threshold of 3 GDPPC per QALY.

Note: All negative ICERS are cost saving. Confidence intervals (95% CI) were estimated as the range between observations at percentiles 2.5 and 97.5 from the Monte Carlo simulations results.

In the threshold analysis, considering that an intervention is cost-effective if its ICER is equal to or less than one GDP per capita per QALY, the minimum required incremental increase in the oxytocin coverage rates to make Uniject a cost-effective strategy ranged from 1.3% in Suriname to 16.2% in Haiti. In 63% of the countries, the required increment was below 5%. Detailed results of the threshold analysis for each country are shown in the second column of Table 3. Base case threshold values as well as the summary results of the sensitivity analysis (95% CI) are also reported in the table.

In the cost-effectiveness analysis, the Uniject strategy ranged from being dominant (i.e. cost saving and health beneficial) to having an ICER of $8,990 per QALY gained. In 26 countries the ICERs were below one GDP per capita and in all countries ICERs were below 3 GDP per capita, showing Uniject as a highly cost-effective strategy.

### Sensitivity analysis

In the deterministic sensitivity analysis, the most influential variables were the discount rate, the estimated increase in oxytocin coverage by using Uniject and the baseline level of oxytocin use. Less influential variables were oxytocin effectiveness to prevent PPH, proportion of maternal deaths due to PPH and cost of Uniject. These findings were consistent among countries. In the PSA, in addition to the aforementioned parameters, we incorporated maternal mortality, PPH baseline risk without oxytocin, probability of severe PPH and unit cost of events. Ranges and probability distributions are shown in Tables 1 and 2.

In Table 3, the 95% CI of the 1,000 results of the threshold values generated in the PSA are reported. Even after considering global parameter uncertainty, the upper bound estimate of the incremental increase in oxytocin use remained below 10% in two-thirds of countries, a value that was considered highly attainable by the expert panel.

**Fig 2:**
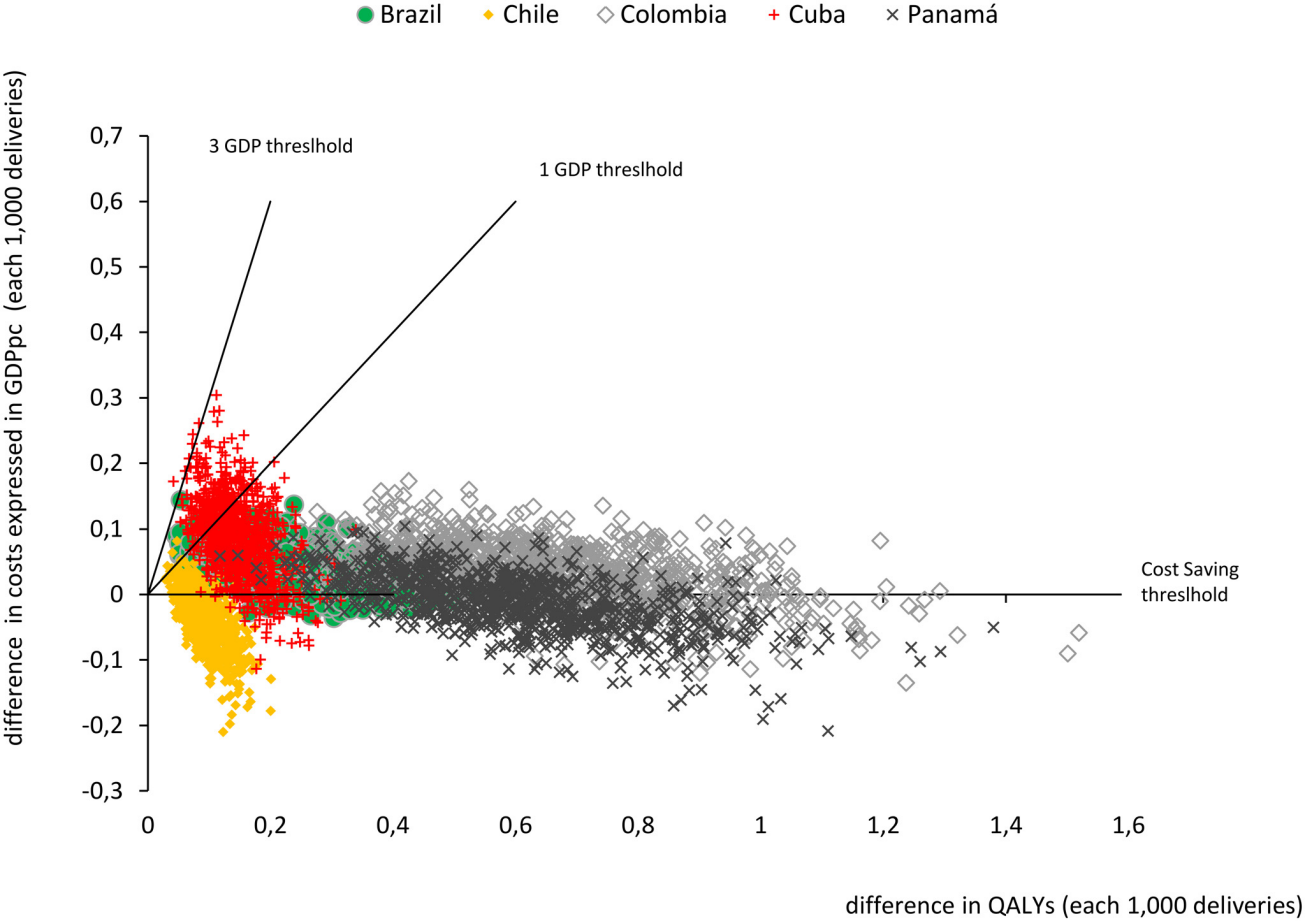
Sensitivity analysis: scatter plot of simulated differences in costs and QALYs between Uniject and current practice of oxytocin in ampoules each 1,000 deliveries in Cuba, Brazil, Chile, Colombia and Panama (origin represents current practice). Notes: GDPpc: Gross Domestic Product per capita; QALYs: Quality Adjusted Life Years. Notes: These five countries were selected to reflect the range of results obtained in the incremental cost-effectiveness ratios (ICERs) in the 30 Latin American and Caribbean countries analysed (percentiles 10%-Cuba, 25%-Colombia, 50%-Brazil, 75%-Panama and 90%-Chile). Each point represents 1 of the 1000 simulated ICERs in the probabilistic sensitivity analysis for each country. Probabilities of being Cost-Saving, or Cost-Effective at 1 and 3 GDPpc for all countries can be seen in Table 3.

In the last columns of Table 3, we show the results of the PSA for the cost-effectiveness analysis, expressed as the likelihood of considering Uniject efficient using three decreasingly stringent criteria (cost saving, cost-effective at a one GDP per capita threshold and cost-effective at a three GDP per capita threshold). With the three GDP thresholds, Uniject would be universally cost-effective in the 30 countries analyzed. In Fig 2, we present the results of the PSA for five countries where it is seen that in the majority of the simulations ICERs were below one GDP.

In the scenario analysis that assumed an incremental cost for Uniject of USD 0.5 (instead of the USD 1.0 of the base case), Uniject was cost saving in 19 countries, with ICERs below 1 GDP per capita in all the 30 countries. In the $1.5 scenario, Uniject remained cost saving in 5 countries and was still cost-effective in 19 countries, considering a 1 GDP per QALY threshold. Only in three countries the ICERs were above 3 GDP per QALY.

## Discussion

In this study, we found that switching to Uniject in 30 LAC countries would increase health and represent both an increment in oxytocin related costs as well as a reduction in the health care costs of PPH and its complications. In 8 out of 30 countries these savings outweighed the excess cost due to the Uniject device, which turned the Uniject strategy cost-saving, and in 26 out of the 30 countries analyzed the ICERs were below one GDP per capita showing Uniject as a highly cost-effective strategy.

### Strengths and Limitations

As a general limitation of our study, we should emphasize the lack of evidence about the effectiveness of Uniject to increase oxytocin utilization rates, a parameter that we had no choice but to estimate through a panel of experts. This specific limitation was tackled by reporting results not only using these effectiveness estimates, but also incorporating the threshold analysis.

Another common limitation in these types of studies is the general lack of accurate epidemiological and cost data in the region. The fact that the analysis included all countries in LAC did not allow us to do a more thorough search and estimation of the whole parameter set for each country. This was addressed by performing a comprehensive sensitivity analysis.

The main strength of our study is that we incorporated an exhaustive evidence base and were able to offer a comprehensive analysis of the entire LAC region.

### Interpretation

The ease of use of Uniject was evaluated in several studies. Tsu et al published two surveys performed in a group of Indonesian and Vietnamese midwives in which 98% and 99% of all respondents stated that Uniject was easier to use and more practical than the conventional use of oxytocin in syringes. Over 96% responded that they preferred Uniject over the regular syringes.[16,17] Althabe et al published a before-and-after quasi experimental study in 2011 in Argentina and 96% of the birth attendants thought that Uniject facilitated the administration of oxytocin.[18]

Moreover, interventions of prophylactic oxytocin to prevent PPH using Uniject in community settings in Ghana and Angola have been proven feasible and effective to reduce PPH.[61,62] However, the potential advantage of using Uniject instead of standard oxytocin to facilitate administration and increase coverage has not been evaluated in any clinical trial yet. One study compared a complex intervention including Uniject and training to increase the use of prophylactic oxytocin in small hospitals in Northern Argentina versus standard of care. A significant increase in the proportion of women who received prophylactic oxytocin was observed in the intervention arm.[18] However, in this study, the intervention was implemented as a package, which impeded to disentangle the specific contribution of Uniject.

Previous economic evaluations showed that Uniject was a cost-effective intervention. In 2006, Seligman et al published an economic evaluation of interventions for reducing PPH in developing countries, including Uniject.[33] All interventions using uterotonics were cost-effective, with very similar costs per DALY. Therefore, they recommended that the selection of interventions should be more related to criteria of access and associated health impacts than related to economic efficiency. But they also reported that oxytocin in Uniject was marginally more cost-effective than oxytocin mono-dose, a result probably influenced by the lower incremental cost the authors assumed for Uniject (USD 0.10). Another study published by Tsu et al, showed that AMTSL could reduce the incidence of PPH without adding much to national health care costs in Vietnam; the cost to avert a case of PPH was $2.10 with standard oxytocin and $4.52 with Uniject.[31] However, so far, there have been no economic evaluations of Uniject oxytocin in LAC that allow concluding that a strategy of using Uniject oxytocin is good value for money, or cost-effective, from the different countries’ perspective.[33]

We intended to evaluate what would be expected in a given health system if standard oxytocin was replaced entirely by Uniject, without mediating any significant educational measure or other specific intervention aimed at increasing the use of oxytocin. However, it could be expected that much more significant increases than those reported in our study could be achieved in the oxytocin coverage rates if other interventions were added along with the introduction of Uniject, as has been observed in other studies. Multifaceted interventions including opinion leaders, academic detailing, reminders, audit and feedback, interactive workshops and training in manual skills have been proven very effective to increase the use of prophylactic oxytocin.[9,18,61]

It is also important to note that there are other potential benefits of Uniject that were not included in our analysis, mainly: reducing infections caused by contaminated needles, decreasing accidental needle injuries, diminishing sharps disposal volume, and preventing syringes and needles re-use. All these potential benefits may have an effect on both clinical and cost outcomes. Additionally, our estimation of current oxytocin coverage rates was based primarily on data from observational studies involving large health facilities, while the situation in the communities or in peripheral health facilities is likely to differ.[14] Therefore, despite the adjustments we made to derive these values, it is not unreasonable to consider they could still be an overestimation of real life oxytocin use. All these factors make our analysis relatively conservative in terms of the potential efficiency of Uniject.

Although we made an effort to incorporate the best available set of parameters for all countries in the region, local realities may differ and parameters may change in the future. Therefore, we made available a web version of the economic model (http://www.iecs.org.ar/iecs-visor.php?cod_producto=811) to enable researchers and health policymakers in each country to fine-tune the model to their specific setting, with the possibility of selecting the values of the different parameters and obtaining new results tailored to the local reality in their country/region.

## Conclusion

Uniject was either cost-saving or very cost-effective in almost all countries in LAC. Even if countries can achieve only small increases in oxytocin use by incorporating Uniject, this strategy could be considered an efficient use of resources. These results showed to be robust in the sensitivity analysis under a wide range of assumptions and scenarios.
