# Correction: Oxytocin in Uniject Disposable Auto-Disable Injection System versus Standard Use for the Prevention of Postpartum Hemorrhage in Latin America and the Caribbean: A Cost-Effectiveness Analysis

**DOI:** 10.1371/journal.pone.0133344

**Published:** 2015-07-15

**Authors:** Andrés Pichon-Riviere, Demián Glujovsky, Osvaldo Ulises Garay, Federico Augustovski, Agustin Ciapponi, Magdalena Serpa, Fernando Althabe

In Table 2, the values in the second column are incorrect. All values should be represented in the thousands. Please see the corrected Table 2 here.

**Table 2:** Country-specific parameters: Base case values, ranges used in sensitivity analysis and source of data.

Country	Annual deliveries [34]	Age at delivery (years)	Skilled birth attendance (%) [35]	Proportion CEmONC (%) [35,36]	Oxytocin use (%) [14,37,38]	Maternal mortality rate (p/100,000) [39]	Proportion of deaths due to PPH (%)	Life expectancy at age of delivery (years) [40]	PPH episode cost (non-severe) US$ 2013	PPH episode cost (severe) US$ 2013	Exch. Rate 2013 (US$ 1) [41]	GDPpp (thousands US$ 2013) [41]
Argentina	694,000	25.5 (23.0–28.1)[42]	99	80^B^	71.1 (57.7–88.4)	77.0 (67.0–87.)	10.00 (5.6–14.9)[43–45]	81.0 (77.0–85.0)	$76.8 (57.6–96.0)^C^	$978.6 (733.9–1,223.2)^C^	$ 5.2	$ 12.0
Bahamas	1,500	21.6 (19.5–23.8)^A^	99	80^B^	71.6 (57.6–89.1)	47.0 (28.0–75.)	16.05 (12.0–20.1)^A^	79.0 (75.0–83.0)	$151.6 (113.7–189.5)^C^	$2,103.7 (1,577.8–2,629.6)^C^	$ 1.0	$ 23.5
Barbados	3,000	21.6 (19.5–23.8)^A^	99	80^B^	71.6 (57.6–89.1)	51.0 (19.0–140.)	9.70 (7.3–12.1)[1]	82.0 (78.0–86.0)	$108.7 (81.5–135.8)^C^	$1,450.5 (1,087.9–1,813.1)^C^	$ 2.0	$ 16.8
Belize	8,000	21.6 (19.5–23.8)^A^	95	80^B^	68.7 (55.3–85.5)	53.0 (33.0–88.)	16.05 (12.0–20.1)^A^	80.0 (76.0–84.0)	$36.1 (27.1–45.1)^C^	$357.9 (268.4–447.4)^C^	$ 2.0	$ 4.6
Bolivia	263,000	21.1 (19.0–23.2)[42]	71	30^B^	51.4 (41.3–63.9)	190.0 (130.0–290.)	15.40 (11.6–19.3)^D^	74.0 (70.0–78.0)	$26.4 (19.8–33.0)^C^	$211.3 (158.4–264.1)^C^	$ 6.7	$ 2.7
Brazil	3,023,000	21.6 (19.5–23.8)[42]	97	80^B^	74.2 (59.6–92.2)	56.0 (36.0–85.)	10.90 (8.2–13.6)[43]	79.0 (75.0–83.0)	$41.8 (31.4–52.3)^C^	$459.3 (344.5–574.1)^C^	$ 1.7	$ 12.3
Chile	241,500	21.6 (19.5–23.8)[42]	99	80^B^	71.6 (57.6–89.1)	25.0 (21.0–29.)	6.53 (4.9–8.2)[2]	84.0 (80.0–88.0)	$96.6 (72.5–120.8)^C^	$1,273.6 (955.2–1,591.9)^C^	$ 516.0	$ 16.3
Colombia	914,000	21.6 (19.4–23.8)[42]	96	80^B^	69.5 (55.9–86.4)	92.0 (80.0–100.)	17.70 (13.3–22.1)^D^	84.0 (80.0–88.0)	$51.2 (38.4–64.0)^C^	$588.7 (441.5–735.9)^C^	$ 1,954.0	$ 8.2
Costa Rica	73,000	21.6 (19.5–23.8)^A^	99	80^B^	71.6 (57.6–89.1)	40.0 (15.0–31.)	15.70 (11.8–19.6)[43]	82.0 (78.0–86.0)	$63.6 (47.7–79.4)^C^	$776.7 (582.5–970.8)^C^	$ 532.3	$ 10.4
Cuba	112,000	21.6 (19.5–23.8)^A^	99	80^B^	71.6 (57.6–89.1)	73.0 (60.0–87.)	4.40 (3.3–5.5)[43]	81.0 (77.0–85.0)	$40.9 (30.7–51.2)^C^	$427.0 (320.3–533.8)^C^	$ 1.0	$ 5.4
Dominican Rep.	216,000	21.6 (19.5–23.8)^A^	98	80^B^	70.9 (57.0–88.2)	115.0 (100.0–210.)	12.65 (9.5–15.8)[43]	80.0 (76.0–84.0)	$41.4 (31.0–51.7)^C^	$438.6 (328.9–548.2)^C^	$ 41.8	$ 5.8
Ecuador	299,000	21.6 (19.5–23.8)^A^	99	80^B^	66.6 (53.6–82.9)	110.0 (62.0–180.)	29.40 (22.1–36.8)[43]	80.0 (76.0–84.0)	$41.5 (31.1–51.9)^C^	$439.1 (329.3–548.8)^C^	$ 1.0	$ 5.6
El Salvador	126,000	21.6 (19.5–23.8)^A^	84	50^B^	60.8 (48.9–75.6)	81.0 (55.0–120.)	16.05 (12.0–20.1)[10]	78.0 (74.0–82.0)	$32.5 (24.4–40.6)^C^	$303.0 (227.3–378.8)^C^	$ 1.0	$ 3.9
Grenada	2,000	21.6 (19.5–23.8)^A^	99	80^B^	71.6 (57.6–89.1)	24.0 (15.0–38.)	16.05 (12.0–20.1)^A^	79.0 (75.0–83.0)	$53.6 (40.2–67.1)^C^	$623.0 (467.3–778.8)^C^	$ 2.7	$ 7.8
Guatemala	467,000	19.9 (17.9–21.9)[46]	51	30^B^	36.9 (29.7–45.9)	120.0 (110.0–140.)	58.10 (43.6–72.6)[11]	78.0 (74.0–82.0)	$28.4 (21.3–35.6)^C^	$246.3 (184.7–307.9)^C^	$ 8.4	$ 3.4
Guyana	14,000	20.7 (18.6–22.8)^A^	83	50^B^	60.1 (48.3–74.7)	280.0 (180.0–430.)	16.05 (12.0–20.1)^A^	70.0 (66.0–74.0)	$32.5 (24.4–40.6)^C^	$300.9 (225.7–376.2)^C^	$ 209.6	$ 3.9
Haiti	266,000	20.1 (18.1–22.1)^A^	26	30^B^	18.8 (15.1–23.4)	315.0 (210.0–610.)	16.05 (12.0–20.1)^A^	70.0 (66.0–74.0)	$17.9 (13.4–22.4)^C^	$87.4 (65.6–109.3)^C^	$ 40.8	$ 0.8
Honduras	203,000	22.2 (20.0–24.4)[42]	67	30^B^	48.5 (39.0–60.3)	100.0 (64.0–160.)	47.06 (35.3–58.8)[12]	79.0 (75.0–83.0)	$24.5 (18.4–30.6)^C^	$182.6 (136.9–228.2)^C^	$ 20.1	$ 2.3
Jamaica	51,000	21.6 (19.5–23.8)^A^	96	80^B^	69.5 (55.9–86.4)	110.0 (77.0–170.)	16.05 (12.0–20.1)^A^	79.0 (75.0–83.0)	$41.3 (31.0–51.6)^C^	$437.7 (328.3–547.1)^C^	$ 91.9	$ 5.6
Mexico	2,217,000	21.6 (19.5–23.8)[42]	94	50^B^	71.6 (57.6–89.1)	50.0 (44.0–56.)	24.30 (18.2–30.4)[43]	80.0 (76.0–84.0)	$74.7 (56.0–93.3)^C^	$941.3 (705.9–1,176.6)^C^	$ 12.3	$ 11.0
Nicaragua	138,000	21.6 (19.5–23.8)[46]	74	30^B^	54.1 (43.5–67.3)	95.0 (54.0–170.)	16.05 (12.0–20.1)[13]	78.0 (74.0–82.0)	$22.5 (16.9–28.1)^C^	$148.7 (111.6–185.9)^C^	$ 24.7	$ 1.8
Panama	70,000	21.6 (19.5–23.8)[42]	91	50^B^	65.9 (52.9–81.9)	92.0 (75.0–110.)	16.40 (12.3–20.5)[43]	82.0 (78.0–86.0)	$72.4 (54.3–90.5)^C^	$904.7 (678.6–1,130.9)^C^	$ 1.0	$ 11.1
Paraguay	156,000	21.6 (19.5–23.8)[42]	97	80^B^	71.1 (57.2–88.5)	99.0 (60.0–160.)	25.40 (19.1–31.8)[43]	80.0 (76.0–84.0)	$35.6 (26.7–44.5)^C^	$355.7 (266.8–444.6)^C^	$ 4,043.7	$ 4.5
Peru	154,000	21.9 (20.0–22.9)[42]	83	50^B^	65.6 (52.7–81.6)	67.0 (42.0–110.)	50.00 (37.5–62.5)[43]	80.0 (76.0–84.0)	$45.7 (34.3–57.2)^C^	$502.8 (377.1–628.5)^C^	$ 2.9	$ 7.1
Saint Lucia	3,000	21.6 (19.5–23.8)^A^	99	80^B^	71.6 (57.6–89.1)	35.0 (22.0–54.)	16.05 (12.0–20.1)^A^	80.0 (76.0–84.0)	$52.5 (39.4–65.6)^C^	$616.9 (462.7–771.2)^C^	$ 2.7	$ 7.6
St. Vincent & Grenadines	2,000	21.6 (19.5–23.8)^A^	99	80^B^	71.6 (57.6–89.1)	48.0 (30.0–78.)	16.05 (12.0–20.1)^A^	78.0 (74.0–82.0)	$47.2 (35.4–59.0)^C^	$537.9 (403.4–672.4)^C^	$ 2.7	$ 6.7
Suriname	10,000	21.6 (19.5–23.8)[42]	90	50^B^	65.1 (52.4–81.0)	130.0 (89.0–190.)	36.00 (27.0–45.0)[43]	81.0 (77.0–85.0)	$61.5 (46.1–76.9)^C^	$749.1 (561.8–936.3)^C^	$ 3.3	$ 9.5
Trinidad & Tobago	20,000	21.6 (19.5–23.8)[42]	98	80^B^	70.9 (57.0–88.2)	46.0 (26.0–84.)	2.80 (2.1–3.5)[43]	76.0 (72.0–80.0)	$122.3 (91.7–152.8)^C^	$1,660.7 (1,245.5–2,075.8)^C^	$ 6.4	$ 20.1
Uruguay	15,000	21.6 (19.5–23.8)[42]	99	80^B^	71.6 (57.6–89.1)	29.0 (21.0–39.)	4.40 (3.3–5.5)[43]	82.0 (78.0–86.0)	$99.1 (74.3–123.9)^C^	$1,315.2 (986.4–1,643.9)^C^	$ 20.0	$ 15.3
Venezuela	158,000	21.6 (19.5–23.8)[42]	95	80^B^	68.7 (55.3–85.5)	92.0 (78.0–110.)	17.01 (12.8–21.3)[2,43] ^D^	81.0 (77.0–85.0)	$69.0 (51.8–86.3)^C^	$858.9 (644.2–1,073.7)^C^	$ 6.5	$ 11.5

Notes:

Abbreviations: Proportion CEmONC: proportion of births at facilities with comprehensive emergency obstetric and newborn care; PPH: postpartum hemorrhage; Exch. Rate: Exchange rate; GDPpp: gross domestic product per capita

**A**: average from other countries (see text)

**B**: estimation based on data from Argentina, Bolivia, El Salvador, and Honduras and references 4 and 5 (see text)

**C**: see methods in manuscript (cost section)

**D:** Data from databases of Ministries of Health (personal communication).

Probability distributions: for the probabilistic sensitivity analysis (PSA) we modeled the variability of Proportion CEmONC (%), Oxytocin use (%) and Proportion of deaths due to PPH (%) using a Beta distribution. Alpha and beta parameters were approximated considering the mean as main estimate and as standard deviation the 10% of the mean value. The costs of severe and non-severe PPH were assumed to follow a Gamma distribution. Alpha and beta parameters were approximated in these cases considering the mean as the main estimate and as standard deviation the 25% of the mean value.

There are errors in Fig 2 where “threshold” is misspelled three times. Please view Fig 2 here.

**Fig 2:**
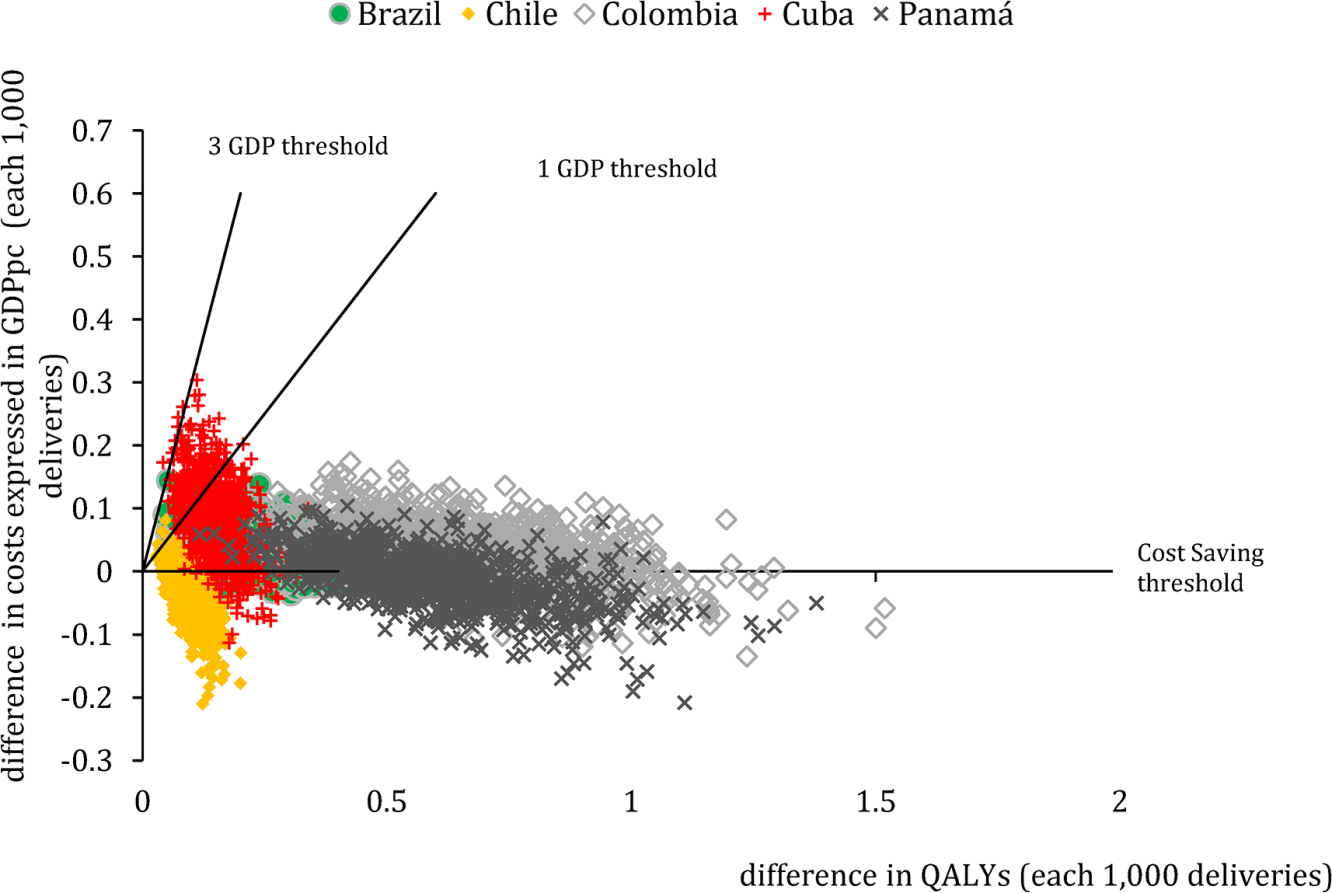
Sensitivity analysis: scatter plot of simulated differences in costs and QALYs between Uniject and current practice of oxytocin in ampoules each 1,000 deliveries in Cuba, Brazil, Chile, Colombia and Panama (origin represents current practice).

Notes: GDPpc: Gross Domestic Product per capita; QALYs: Quality Adjusted Life Years. Notes: These five countries were selected to reflect the range of results obtained in the incremental cost-effectiveness ratios (ICERs) in the 30 Latin American and Caribbean countries analysed (percentiles 10%-Cuba, 25%-Colombia, 50%-Brazil, 75%-Panama and 90%-Chile). Each point represents 1 of the 1000 simulated ICERs in the probabilistic sensitivity analysis for each country. Probabilities of being Cost-Saving, or Cost-Effective at 1 and 3 GDPpc for all countries can be seen in Table 2.
